# Supplementary material for: Human motor learning dynamics in high-dimensional tasks
Source: PLoS Comput Biol. 2024 Oct 14;20(10):e1012455. doi: 10.1371/journal.pcbi.1012455 (PMC11501022; doi:10.1371/journal.pcbi.1012455)
Supplement: S1 File — Document containing supplementary HML model details, its convergence analysis, and additional model-based investigation into human motor learning behavior results. (PDF) [file pcbi.1012455.s001.pdf]

Human motor learning dynamics in high-dimensional tasks: Supporting information

Ankur Kamboj[1*], Rajiv Ranganathan[2], Xiaobo Tan[1], Vaibhav Srivastava[1]

1 Department of Electrical and Computer Engineering, Michigan State University, East Lansing, Michigan, United States of America
2 Department of Kinesiology, Michigan State University, East Lansing, Michigan, United States of America

* ankurank@msu.edu

1 Model

1.1 Human perception model of BoMI

The BoMI mapping

$$\dot{\boldsymbol{x}} = C\boldsymbol{u} \tag{1}$$

is described in terms of finger joint velocities $\boldsymbol{u}$ and cursor velocities $\dot{\boldsymbol{x}}$. However, we hypothesize that humans interacting with the BoMI perceive these velocities as increments in cursor positions and finger joint angles. Consequently, we adopt a standard technique from adaptive control that allows us to rewrite the BoMI mapping equation using filtered joint and cursor position data [1]. We write the dynamics (Eq (1)) using a mixed frequency-time notation as $s\boldsymbol{x} = C\boldsymbol{u}$, where s denotes the Laplace (frequency) variable. Dividing both sides by $s + a$, for some sufficiently large $a > 0$, yields

$$\frac{s}{s+a}\boldsymbol{x} = -\frac{a}{s+a}\boldsymbol{x} + \boldsymbol{x} = \frac{1}{s+a}C\boldsymbol{u}. \tag{2}$$

Define the signals filtered cursor position $\boldsymbol{\chi} = \boldsymbol{x}/(s + a)$, and filtered change in hand joint angles $\delta\boldsymbol{q} = \boldsymbol{u}/(s + a)$. Equivalently

$$\begin{aligned} \dot{\boldsymbol{\chi}} &= -a\boldsymbol{\chi} + \boldsymbol{x}, \\ \dot{\delta\boldsymbol{q}} &= -a\delta\boldsymbol{q} + \boldsymbol{u} + \boldsymbol{\xi}_q, \end{aligned} \tag{3}$$

where we add the perceptual noise $\boldsymbol{\xi}_q$ to capture the inaccuracies in the filtering process. Then, in the time-domain (Eq (2)) reduces to $-a\boldsymbol{\chi} + \boldsymbol{x} = C\delta\boldsymbol{q}$. Defining $\delta\boldsymbol{x} = -a\boldsymbol{\chi} + \boldsymbol{x}$, we get the system equation as

$$\delta\boldsymbol{x} = C\delta\boldsymbol{q}. \tag{4}$$

$\delta\boldsymbol{x}$ and $\delta\boldsymbol{q}$ are termed *filtered increments in cursor positions*, and *filtered increments in joint angles*, respectively.

1.2 The inverse learning model

We postulate that the participant determines the joint angle velocities through gradient-based learning on a regularized quadratic error function,

$$\dot{\boldsymbol{u}} = -\eta \nabla_{\boldsymbol{u}} \left(\frac{1}{2} \left\| \dot{\hat{\boldsymbol{x}}} - k_P \boldsymbol{e}_{\boldsymbol{x}} \right\|^2 + \frac{\mu}{2} \left\| \boldsymbol{u} \right\|^2 \right) + \boldsymbol{\xi}_u, \tag{5}$$

where $\boldsymbol{u} \in \mathbb{R}^m$, $\hat{\boldsymbol{x}}$ is the estimated cursor position based on the participant's estimate of mapping matrix $\hat{C}$, $\eta > 0$ is the inverse learning rate, and $\boldsymbol{\xi}_u$ is the exploration noise.

Upon simplification, the gradient in (Eq (5)),

$$\begin{aligned}
&\nabla_{\boldsymbol{u}} \left(\frac{1}{2} \left\| \dot{\hat{\boldsymbol{x}}} - k_P \boldsymbol{e}_{\boldsymbol{x}} \right\|^2 + \frac{\mu}{2} \left\| \boldsymbol{u} \right\|^2 \right) \\
&\quad = \nabla_{\boldsymbol{u}} \left(\frac{1}{2} \left\| \hat{C}\boldsymbol{u} - k_P \boldsymbol{e}_{\boldsymbol{x}} \right\|^2 + \frac{\mu}{2} \left\| \boldsymbol{u} \right\|^2 \right) \\
&\quad = \hat{C}^\top (\hat{C}\boldsymbol{u} - k_P \boldsymbol{e}_{\boldsymbol{x}}) + \mu \boldsymbol{u} \\
&\quad = (\hat{C}^\top \hat{C} + \mu I)\boldsymbol{u} - k_P \hat{C}^\top \boldsymbol{e}_{\boldsymbol{x}},
\end{aligned} \tag{6}$$

which results in

$$\begin{aligned}
\dot{\boldsymbol{u}} &= -\eta \left((\hat{C}^\top \hat{C} + \mu I)\boldsymbol{u} - k_P \hat{C}^\top \boldsymbol{e}_{\boldsymbol{x}} \right) + \boldsymbol{\xi}_u \\
&= -\eta \left((\Phi^\top \hat{W}^\top \hat{W} \Phi + \mu I)\boldsymbol{u} - k_P \Phi^\top \hat{W}^\top \boldsymbol{e}_{\boldsymbol{x}} \right) + \boldsymbol{\xi}_u.
\end{aligned} \tag{7}$$

1.3 Sufficiently rich perceptual noise

Definition 1 (Sufficiently Rich Signal). *A stationary signal $\omega(t)$ is called sufficiently rich of order n if the support of the spectral measure of ω, defined by,*

$$\mathcal{S}_\omega(\theta) = \int_{-\infty}^{\infty} e^{-j\theta\tau} \mathcal{R}_\omega(\tau) \mathrm{d}\tau,$$

contains at least n points, where $\mathcal{R}_\omega(\tau) = \lim_{T\to\infty} \frac{1}{T} \int_0^T \omega(t)\omega(t+\tau)\mathrm{d}t$ is the autocovariance of ω [2]*.*

The perceptual noise $\boldsymbol{\xi}_q$ in the filtered increment in joint angle dynamics (Eq (3)) is a sufficiently rich noise satisfying Definition 1.

Remark 1. *For purposes of simulation and model fitting, $\boldsymbol{\xi}_q$ is treated as white noise with intensity σ_q. $\boldsymbol{\xi}_q$ is a stationary signal as per [2, Definition 5.2.2], with a flat spectral power density $\mathcal{S}_\omega(\theta)$. $\boldsymbol{\xi}_q$ is thus sufficiently rich of any order.*

2 Analysis of adaptive control-based HML model

This section is dedicated to the analysis of the following proposed model of HML from a control-theoretic perspective.

$$\dot{\delta\boldsymbol{q}} = -a\delta\boldsymbol{q} + \boldsymbol{u} + \boldsymbol{\xi}_q \tag{8a}$$

$$\dot{\tilde{C}} = -\gamma \tilde{C} \delta\boldsymbol{q} \delta\boldsymbol{q}^\top \tag{8b}$$

$$\dot{\boldsymbol{e}}_x = -C\boldsymbol{u} \tag{8c}$$

$$\dot{\boldsymbol{u}} = -\eta \left((\hat{C}^\top \hat{C} + \mu I)\boldsymbol{u} - k_P \hat{C}^\top \boldsymbol{e}_{\boldsymbol{x}} \right) + \boldsymbol{\xi}_u, \tag{8d}$$

where $\tilde{C} = \hat{C} - C$, and we have used $\dot{\tilde{C}} = \dot{\hat{C}}$ We aim to show that the mathematical model of human forward and inverse learning proposed here, under certain viable assumptions, evolves in a stable fashion and converges to equilibrium exponentially.

The motor learning literature suggests that the forward learning dynamics evolve on a slower timescale than the inverse learning dynamics (refer to the Discussion section in the main text). Consistent with the literature, our parameter fits suggest $\gamma \ll \eta, k_P$, i.e., $\tilde{C}$ dynamics evolve on a slower timescale, and $\boldsymbol{u}, \boldsymbol{e_x}$ dynamics evolve on a faster timescale.

Decomposing (Eq (1)) and $\widehat{\delta \boldsymbol{x}} = \hat{C}\delta\boldsymbol{q}$ into n equations (one for each cursor motion axis), we obtain $\widehat{\delta \boldsymbol{x}}_i = \hat{C}_i \delta\boldsymbol{q}$ for all $i \in \{1, \ldots, n\}$. A similar expression can be written for (Eq (1)). We analyze these equations separately, and for ease of notation, we will drop the index i.

Defining the slow variable $\bar{e}_x = k_P e_x \in \mathbb{R}$, we get the modified reaching error dynamics as

$$\dot{\bar{e}}_x = k_P(\dot{x}^{\text{des}} - \dot{x}) = -k_P C\boldsymbol{u}. \tag{9}$$

Let $f : \mathbb{R}^m \times \mathbb{R}^{1\times m} \mapsto \mathbb{R}^{1\times m},\ g_1 : \mathbb{R}^m \times \mathbb{R} \times \mathbb{R}^{1\times m} \mapsto \mathbb{R}^m,\ g_2 : \mathbb{R}^m \mapsto \mathbb{R}$ be defined by

$$\begin{aligned} f(\tilde{C}) &= -\,\tilde{C}\delta\boldsymbol{q}\delta\boldsymbol{q}^\top, \\ g_1(\boldsymbol{u}, \bar{e}_x, \hat{C}) &= -\,((\hat{C}^\top\hat{C} + \mu I)\boldsymbol{u} - \hat{C}^\top \bar{e}_x), \\ g_2(\boldsymbol{u}) &= -\,C\boldsymbol{u}. \end{aligned}$$

Then the HML model dynamics in (Eqs (8b), (8d), (9)) can be equivalently written as

$$\dot{\hat{C}} = \gamma\ f(\tilde{C}), \tag{10a}$$

$$\dot{\boldsymbol{u}} = \eta\ g_1(\boldsymbol{u}, \bar{e}_x, \hat{C}) + \boldsymbol{\xi}_u \tag{10b}$$

$$\dot{\bar{e}}_x = k_P\ g_2(\boldsymbol{u}), \tag{10c}$$

where we treat $\delta\boldsymbol{q}$ as an exogenous input for the purpose of analysis. Rewriting system (Eq (10)) in the new timescale $t \mapsto \gamma t$, we get the singularly perturbed system defined by

$$\dot{\tilde{C}} = f(\tilde{C}), \tag{11a}$$

$$\varepsilon\dot{\boldsymbol{\rho}}_u = \varepsilon \begin{bmatrix} \dot{\boldsymbol{u}} \\ \dot{\bar{e}}_x \end{bmatrix} = \begin{bmatrix} \varepsilon_e g_1(\boldsymbol{u}, \bar{e}_x, \tilde{C}) + \varepsilon\boldsymbol{\xi}_u/\gamma \\ \varepsilon_u g_2(\boldsymbol{u}) \end{bmatrix}, \tag{11b}$$

where $\varepsilon = \varepsilon_u\varepsilon_e$, $\varepsilon_u = \gamma/\eta$, and $\varepsilon_e = \gamma/k_P$. $\varepsilon = \gamma^2/\eta k_P \ll 1$ and thus $\boldsymbol{u}, \bar{e}_x$ are the fast variables.

It can be verified that under the persistency of excitation of $\delta\boldsymbol{q}$, $(\boldsymbol{u}, \bar{e}_x, \tilde{C}) = (0, 0, 0)$ is an isolated equilibrium of system (Eq (11)) in the absence of exploration noise $\boldsymbol{\xi}_u$. Moreover, the functions f, g_1, g_2 are locally Lipschitz, and their partial derivatives up to the second-order are bounded in their respective domains containing the origin.

2.1 Slower-timescale forward learning dynamics

The reduced system associated with (Eq (11)) is

$$\dot{\tilde{C}} = f(\tilde{C}) = -\tilde{C}\delta\boldsymbol{q}\delta\boldsymbol{q}^\top, \tag{12}$$

where the fast-varying states are settled at the isolated equilibrium $(\boldsymbol{u},\, \bar{e}_x) = (0, 0)$.

Lemma 1 (***Stability of the Reduced System (Eq** (12)**)***). *The solution of the reduced system (Eq (12)) converges globally exponentially to the origin.*

Proof. $\delta\boldsymbol{q}$ dynamics with added sufficiently rich noise $\boldsymbol{\xi}_q$ at the equilibrium value of fast-varying states is

$$\dot{\delta\boldsymbol{q}} = -a\delta\boldsymbol{q} + \boldsymbol{\xi}_q,$$
$$\text{or equivalently, } \delta\boldsymbol{q} = \frac{1}{s+a}\boldsymbol{\xi}_q. \tag{13}$$

Invoking [3, Theorem 4.2], with $Q(s) = 1$ and $p(s) = s + a$, $\delta\boldsymbol{q}$ is persistently exciting. The lemma is then proved using standard adaptive control techniques [2], which leverage the radially unbounded Lyapunov function $V_r(\tilde{C}) = \frac{1}{2}\tilde{C}\tilde{C}^\top$ and persistency of excitation of $\delta\boldsymbol{q}$. □

2.2 Faster-timescale inverse learning dynamics

Ignoring the noise term $\boldsymbol{\xi}_u$ in $\boldsymbol{u}$ dynamics, the boundary layer system associated with (Eq (11)) is defined in the new timescale $\tau_u = t/\varepsilon$ as

$$\frac{d\boldsymbol{\rho}_u}{d\tau_u} = \begin{bmatrix} \frac{d\bar{\boldsymbol{u}}}{d\tau_u} \\ \frac{d\bar{e}_x}{d\tau_u} \end{bmatrix} = \begin{bmatrix} -1/\varepsilon_u \left((\hat{C}^\top\hat{C} + \mu I)\bar{\boldsymbol{u}} - \hat{C}\bar{e}_x\right) \\ -1/\varepsilon_e(C\bar{\boldsymbol{u}}) \end{bmatrix}, \tag{14a}$$

where $\boldsymbol{u}$ is replaced by noise-free joint velocities $\bar{\boldsymbol{u}}$, and the slow-varying state $\hat{C} = \tilde{C} + C$ is frozen in time.

Lemma 2 (***Stability of the Boundary Layer System (Eq** (14)**)***). *For the boundary layer system (Eq* (14)*), the equilibrium point at the origin is globally exponentially stable, given that the frozen state $\tilde{C}$ satisfies $\left\|\tilde{C}\right\| < \min\{\mu, \theta_c\}$, where $\theta_c \geq 0$ is the largest value that satisfies the cubic inequality* $\theta_c^3 - \theta_c^2\left(2\|C\| + 1\right) + \theta_c\left(\|C\|^2 + 2\|C\| + \mu\right) - \|C\|^2 < 0.$

Proof. Consider the radially unbounded Lyapunov function

$$\begin{aligned} V_b(\bar{\boldsymbol{u}}, \bar{e}_x) &= \frac{\varepsilon_u}{2}(\bar{\boldsymbol{u}} - \boldsymbol{u}^*)^\top(\bar{\boldsymbol{u}} - \boldsymbol{u}^*) + \varepsilon_e\bar{e}_x^2, \\ &= \frac{1}{2}\boldsymbol{\rho}_u^\top P\boldsymbol{\rho}_u, \end{aligned}$$

where $\boldsymbol{u}^* = \bar{C}^{-1}\hat{C}^\top\bar{e}_x$, $\boldsymbol{\rho}_u = [\bar{\boldsymbol{u}}^\top, \bar{e}_x]^\top$,
$P = \begin{bmatrix} \varepsilon_u I & -\varepsilon_u\bar{C}^{-1}\hat{C}^\top \\ -\varepsilon_u\hat{C}\bar{C}^{-1} & (2\varepsilon_e + \varepsilon_u\hat{C}\bar{C}^{-1}\bar{C}^{-1}\hat{C}^\top) \end{bmatrix} \succ \mathbf{0}$, and $\bar{C} = (\hat{C}^\top\hat{C} + \mu I)$. We thus have $\lambda_{\min}(P)\|\boldsymbol{\rho}_u\|^2 \leq V_b(\bar{\boldsymbol{u}}, \bar{e}_x) \leq \lambda_{\max}(P)\|\boldsymbol{\rho}_u\|^2$, where $\lambda_{\min}(\cdot)$ and $\lambda_{\max}(\cdot)$ are the minimum and maximum eigenvalues of the matrix in the argument.

The time-derivative ($\frac{d}{d\tau_u}$) of V_b along the boundary layer system trajectories (Eq (14)) is

$$\begin{aligned} \frac{dV_b}{d\tau_u} &= \varepsilon_u(\bar{\boldsymbol{u}} - \boldsymbol{u}^*)^\top\dot{\bar{\boldsymbol{u}}} + 2\varepsilon_e\bar{e}_x\dot{\bar{e}}_x, \\ &= -(\bar{\boldsymbol{u}} - \boldsymbol{u}^*)^\top(\bar{C}\bar{\boldsymbol{u}} - \hat{C}^\top\bar{e}_x) - 2\bar{e}_xC\bar{\boldsymbol{u}}, \\ &= -\bar{\boldsymbol{u}}^\top\bar{C}\bar{\boldsymbol{u}} + \bar{\boldsymbol{u}}^\top\hat{C}^\top\bar{e}_x + \bar{e}_x\hat{C}\bar{C}^{-1}\bar{C}\bar{\boldsymbol{u}} - \bar{e}_x\hat{C}\bar{C}^{-1}\hat{C}^\top\bar{e}_x - 2\bar{e}_xC\bar{\boldsymbol{u}}, \\ &= -\bar{\boldsymbol{u}}^\top\bar{C}\bar{\boldsymbol{u}} - \bar{e}_x\hat{C}\bar{C}^{-1}\hat{C}^\top\bar{e}_x + \bar{e}_x(2\hat{C} - 2C)\bar{\boldsymbol{u}}, \\ &= -\boldsymbol{\rho}_u^\top Q\boldsymbol{\rho}_u + 2\bar{e}_x\tilde{C}\bar{\boldsymbol{u}}, \\ &\leq -\lambda_{\min}(Q)\|\boldsymbol{\rho}_u\|^2 + 2\|\bar{e}_x\|\left\|\tilde{C}\right\|\|\bar{\boldsymbol{u}}\|, \end{aligned} \tag{15}$$

where, $Q = \begin{bmatrix} \bar{C} & 0 \\ 0 & \hat{C}\bar{C}^{-1}\hat{C}^\top \end{bmatrix} \succ \mathbf{0}$. Using the fact that $\|\boldsymbol{\rho}_u\|^2 = \|\bar{\boldsymbol{u}}\|^2 + \|\bar{e}_x\|^2$, we get

$$\frac{dV_b}{d\tau_u} \leq -\lambda_{\min}(Q)\left(\|\bar{\boldsymbol{u}}\|^2 + \|\bar{e}_x\|^2\right) + 2\|\bar{e}_x\|\left\|\tilde{C}\right\|\|\bar{\boldsymbol{u}}\| = -\boldsymbol{\psi}(\bar{\boldsymbol{u}}, \bar{e}_x)^\top \Lambda \boldsymbol{\psi}(\bar{\boldsymbol{u}}, \bar{e}_x), \tag{16}$$

where $\boldsymbol{\psi}(\bar{\boldsymbol{u}}, \bar{e}_x) = [\|\bar{\boldsymbol{u}}\|, \|\bar{e}_x\|]^\top$, and $\Lambda = \begin{bmatrix} \lambda_{\min}(Q) & -\left\|\tilde{C}\right\| \\ -\left\|\tilde{C}\right\| & \lambda_{\min}(Q) \end{bmatrix}$.

Note that $\lambda_{\min}(Q) = \min\{\lambda_{\min}(\bar{C}), \hat{C}\bar{C}^{-1}\hat{C}^\top\}$, where $\lambda_{\min}(\bar{C}) = \mu$. Since $\hat{C} = C + \tilde{C}$, we have $\|C\| - \left\|\tilde{C}\right\| \leq \left\|\hat{C}\right\|$, which gives us

$$\begin{aligned} \hat{C}\bar{C}^{-1}\hat{C}^\top &= \hat{C}(\hat{C}^\top\hat{C} + \mu I)^{-1}\hat{C}^\top = \hat{C}\hat{C}^\top(\hat{C}\hat{C}^\top + \mu)^{-1} \\ &= \frac{\left\|\hat{C}\right\|^2}{\left\|\hat{C}\right\|^2 + \mu} \geq \frac{\left(\|C\| - \left\|\tilde{C}\right\|\right)^2}{\left(\|C\| - \left\|\tilde{C}\right\|\right)^2 + \mu} > 0. \end{aligned}$$

Since $\lambda_{\min}(Q) > 0$, and Λ is a symmetric matrix, it is positive definite if $\left\|\tilde{C}\right\| < \lambda_{\min}(Q) = \min\left\{\mu, \frac{(\|C\| - \|\tilde{C}\|)^2}{(\|C\| - \|\tilde{C}\|)^2 + \mu}\right\}$. The second bound gives us $\left\|\tilde{C}\right\| < \frac{(\|C\| - \|\tilde{C}\|)^2}{(\|C\| - \|\tilde{C}\|)^2 + \mu}$, which is always satisfied for the largest value θ_c that satisfies the cubic inequality

$$\theta_c^3 - \theta_c^2(2\|C\| + 1) + \theta_c\left(\|C\|^2 + 2\|C\| + \mu\right) - \|C\|^2 < 0. \tag{17}$$

Therefore, if $\left\|\tilde{C}\right\| \leq \min\{\mu, \theta_c\}$ then $\frac{dV_b}{d\tau_u}$ is negative definite, and the boundary layer system is globally exponentially stable.

□

Remark 2. *Inequality* (17) *is satisfied for* $\theta_c = 0$. *By continuity of the cubic equation in* θ_c, *there exists a non-zero measure set where this inequality is satisfied.*

2.3 Coupled forward-inverse motor learning dynamics

We now show the stability and convergence of our proposed HML model using singular perturbation arguments. Define $\Omega_c = \left\{\left(\tilde{C}, \bar{\boldsymbol{u}}, \bar{e}_x\right) \in \mathbb{R}^{1\times m} \times \mathbb{R}^m \times \mathbb{R} \middle| \left\|\tilde{C}\right\| \leq \delta_c\right\}$, where $\delta_c = \min\{\mu, \theta_c\}$, and θ_c is the largest value that satisfies the cubic inequality (Eq (17)).

Theorem 1 (***Stability of the HML Model*** (Eq (10)))**.** *In the absence of exploration noise* $\boldsymbol{\xi}_u$, *there exists sufficiently small* ε^* *such that for all* $\gamma^2/\eta k_P \in (0, \varepsilon^*)$, Ω_c *is positively invariant, and for all* $\left(\tilde{C}(0), \bar{\boldsymbol{u}}(0), \bar{e}_x(0)\right) \in \Omega_c$, *the trajectories of the proposed HML model in Eq* 9 *exponentially converge to the origin.*

Proof. From the proof of Lemma 1, we have $\left\|\tilde{C}(t)\right\| \leq e^{-\gamma\lambda_{\min}(\delta\boldsymbol{q}\delta\boldsymbol{q}^\top)t}\left\|\tilde{C}(0)\right\| \leq \left\|\tilde{C}(0)\right\|$, implying $\left\|\tilde{C}(t)\right\|$ is non-increasing. Thus, $\left\|\tilde{C}(0)\right\| < \delta_c = \min\{\mu, \theta_c\}$ implies $\left\|\tilde{C}(t)\right\| < \delta_c$ for all $t > 0$. Therefore, from Lemma 2, the boundary layer system is globally exponentially stable. Using similar techniques as in [4, Theorem 11.4], the exponential stability of the system can be proved by constructing a composite Lyapunov function using the Lyapunov functions $V_r(\tilde{C})$ and $V_b(\bar{\boldsymbol{u}}, \bar{e}_x)$, for $(\tilde{C}, \bar{\boldsymbol{u}}, \bar{e}_x)$ starting in the set Ω_c. □

Remark 3. *We show exponential convergence of HML model states (Eq* (10)*) to the origin in the absence of exploration noise in Theorem 1. With the stochastic exploration noise* $\boldsymbol{\xi}_u$ *added to the model, using ideas similar to [5, Section 8.6], it can be shown that the HML model trajectories enter and stay in a ball of radius* $O(|\sigma_u|)$ *centered around the origin.*

3 HML model-based investigation into motor learning behavior

3.1 Exploration versus exploitation trade-off

Apart from the inverse learning parameter η which had the most significant effect on the exploration versus exploitation trade-off, we also show how the exploration noise intensity affects this performance metric in Fig 1.

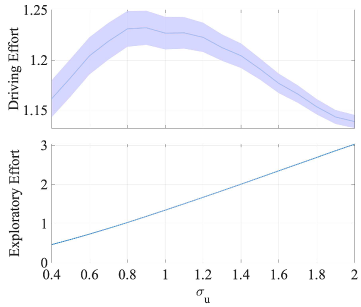

Fig 1. Effort variation with σ_u. Distribution of driving and exploratory effort across trials as σ_u is varied around its fit value 0.8764. Driving effort is highest around the fitted value of σ_u ($p < 0.01$), while exploratory effort increases monotonically with σ_u.

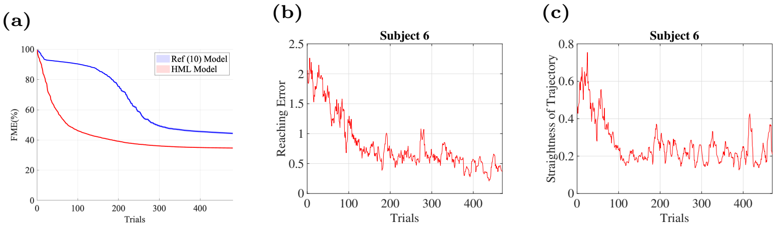

Fig 2. Comparing HML model with Ref [6] model. (a) Comparing the `FME` curves from the model in Ref [6] to the HML model shows that the model in Ref [6] is not as accurate as HML model in capturing the human skill state (represented by the participant's estimate of BoMI mapping matrix $\hat{C}$) for this motor learning task. (b) and (c) show the `RE` and `SoT` evolution curves for Subject 6.

3.2 Comparative analysis of HML model's efficacy in explaining the motor learning

Fig 2 shows the evolution of `FME` from the HML model and the model in Ref [6], both fitted on the experimental data of Subject 6. Looking at Fig 2, `FME` from our proposed model aligns more closely to the `RE` and `SoT` observed from the experimental data for Subject 6 as compared to the `FME` from the Ref [6] model. There's a sharp initial decrease in `FME` out of HML model Fig 2, and also in the `RE` and the `SoT` Fig 2c, 2b for

Subject 6. Whereas, `FME` out of the Ref [6] shows a slow initial decrease, followed by a fast decay much later in the game trials.

References

1. Sastry S, Bodson M. Adaptive Control: Stability, Convergence, and Robustness. Prentice-Hall, Inc.; 1989.

2. Ioannou PA, Sun J. Robust Adaptive Control. vol. 1. PTR Prentice-Hall Upper Saddle River, NJ; 1996.

3. Mareels IM, Gevers M. Persistency of excitation criteria for linear, multivariable, time-varying systems. Mathematics of Control, Signals and Systems. 1988;1:203–226.

4. Khalil HK. Nonlinear Systems. 3rd ed. Prentice Hall; 2002.

5. Kushner HJ. Introduction to Stochastic Control. Holt, Rinehart and Winston New York; 1971.

6. Pierella C, Casadio M, Mussa-Ivaldi FA, Solla SA. The dynamics of motor learning through the formation of internal models. PLoS Computational Biology. 2019;15(12):e1007118.
