# Supplementary figures and images for: Human motor learning dynamics in high-dimensional tasks
Source: PLoS Comput Biol. 2024 Oct 14;20(10):e1012455. doi: 10.1371/journal.pcbi.1012455 (PMC11501022; doi:10.1371/journal.pcbi.1012455)

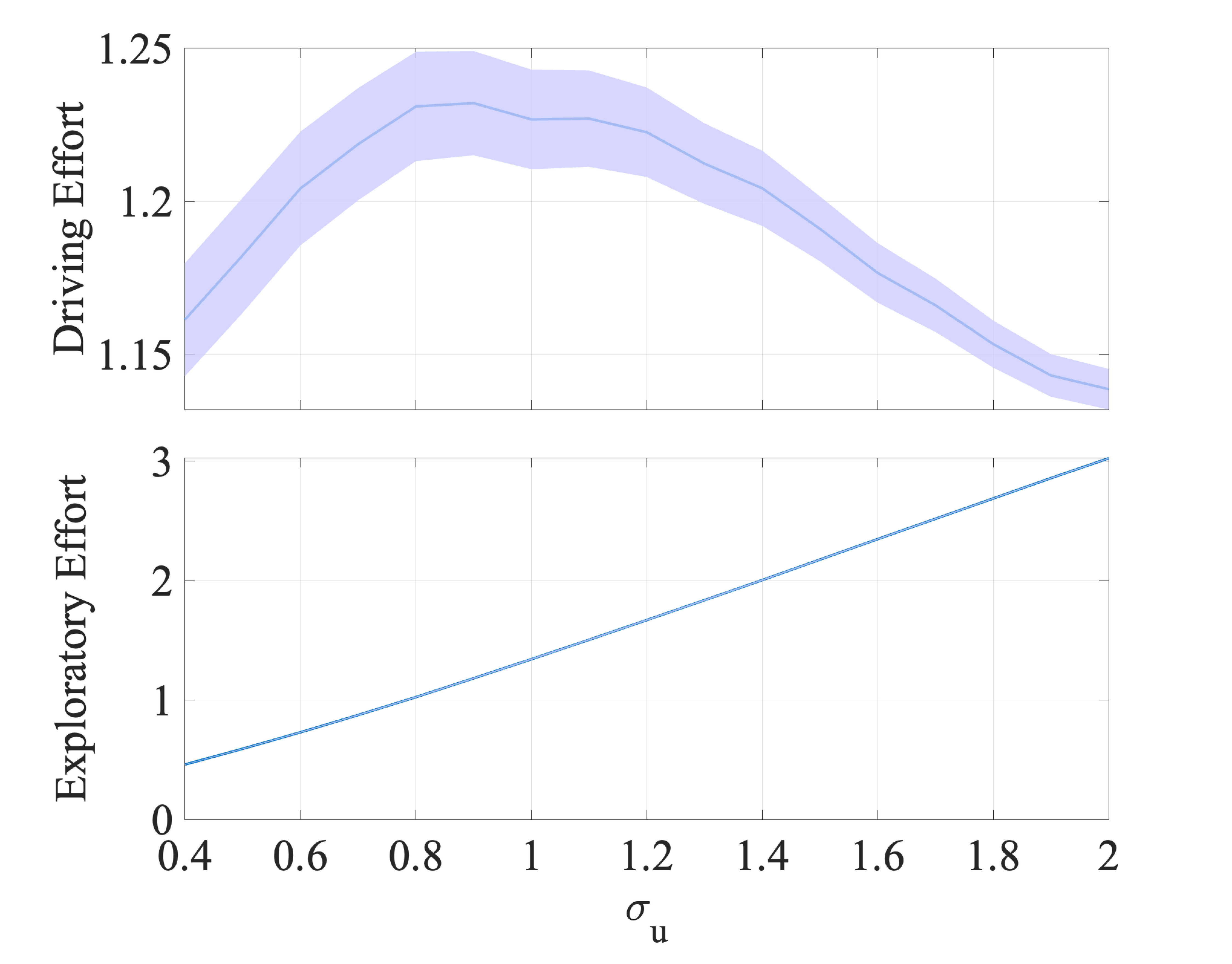

Supplement: S1 Fig — Distribution of driving and exploratory effort across trials as σu is varied around its fit value 0.8764. Driving effort is highest around the fitted value of σu (p < 0.01), while exploratory effort increases monotonically with σu. (TIF) [file pcbi.1012455.s002.tif]
