# Supplementary material for: Human motor learning dynamics in high-dimensional tasks
Source: PLoS Comput Biol. 2024 Oct 14;20(10):e1012455. doi: 10.1371/journal.pcbi.1012455 (PMC11501022; doi:10.1371/journal.pcbi.1012455)
Supplement: Attachment — Submitted filename: PLoS_SOR.pdf [file pcbi.1012455.s003.pdf]

Barbara Webb
Academic Editor
PLOS Computational Biology

Andrea E. Martin
Section Editor
PLOS Computational Biology

Ankur Kamboj
Electrical and Computer Engineering
Michigan State University
East Lansing, MI 48823, USA
email: ankurank@msu.edu

East Lansing, July 19, 2024

Re: Paper PCOMPBIOL-D-24-00667: *Human motor learning dynamics in high-dimensional tasks*

Dear Dr. Barbara Webb and Dr. Andrea E. Martin,

Please find enclosed the revision of our manuscript number PCOMPBIOL-D-24-00667, submitted as a journal paper in the *PLoS Computational Biology*.

We would like to thank you all for the patience and effort in the reviewing process of our paper. We appreciate the thoughtful and constructive comments from the reviewers on how to improve the paper. In the following pages, we provide a detailed list of the changes made in the revised version according to the reviewers' observations.

We look forward to hearing from you. Sincerely yours,

Ankur Kamboj
Rajiv Ranganathan
Xiaobo Tan
Vaibhav Srivastava

Statement of Revision

In the following, we provide a detailed account of all the changes that we have made in the revised version of the paper. We have structured this list in separate blocks, corresponding to the suggestions made by the reviewers. We would like to thank all the reviewers for their thoughtful and constructive remarks. To aid in reviewing the revised manuscript, all changes to the manuscript have been made in blue typeface.

Comments by Reviewer # 1

[R1: 1] "This work represents a significant advance in the understanding of motor learning by providing a computational framework that represents continuous learning, a much more ecologically relevant form of learning than the forms of motor learning that have been addressed in countless previous studies."

We thank the reviewer for the kind comments. We highly appreciate them for taking the time to review our manuscript and suggest possible points of improvements. We have tried to address the concerns and comments to the best of our abilities, and hope that manuscript is clearer and in a better shape now.

[R1: 2] "Limitations of the approach include the narrow database used for the empirical application of the model (data from a previously published study) and the use of only one learning paradigm."

We appreciate the reviewer's comment and acknowledge these concerns. The proposed model is capable of explaining motor learning behaviors more broadly, and we believe the learning paradigm we used is quite extensive for several reasons. Firstly, the experiment involves learning to control a high number of degrees of freedom mapped to a low-dimensional task, which is a critical factor in many motor learning tasks. Secondly, the use of kinematic "synergies" for hand movements has been well documented [4], enabling the task to capture various features of the model effectively. We also recognize that the model was tested on a limited set of participants. However, because the focus was on learning, each participant contributed a large number of data points. We are planning future experiments to test the model's predictions more extensively. We have now acknowledged this in the section 'Discussion: Future directions' (page 14) as follows-

"The proposed model is capable of explaining motor learning behaviors more broadly, and we believe the experimental paradigm we used is quite extensive for several reasons. Firstly, the experiment involves learning to control a high number of degrees of freedom mapped to a low-dimensional task, which is a critical factor in many motor learning tasks. Secondly, the use of kinematic "synergies" for hand movements has been well documented [4], enabling the task to capture various features of the model effectively. We plan to test the generality of model on a broader set of learning paradigms going forward."

[R1: 3] "A possible suggestion for improving the manuscript would be to indicate what strong predictions are made for future empirical testing of the framework."

We thank the reviewer for this comment and suggestions on improving the manuscript. We have expanded upon 'Future directions' to include some possible experimental setups that can be used to test various motor learning phenomena under the current experimental paradigm, specifically satisficing and flexibility versus performance. Following are the changes we have included in the section 'Discussion: Future directions' (page 14)-

"Furthermore, we can generate hypotheses to experimentally validate various motor learning trade-offs. For example, our model suggests the satisficing behavior during motor learning. This can be empirically evaluated by varying the target sizes ρ_x and examining the relationship between the success probabilities and learning threshold levels. Various metrics can be used as a measure of learning threshold level of a

participant, such as straightness of trajectory, reaching error, etc. Another prediction of the model is that on a constrained learning time, using lower number of synergies should lead to a more robust learning performance. A possible experiment could be to evaluate the minimum number of synergies employed by a particular participant to learn the task if we keep perturbing/changing the mapping matrix periodically during the experiment. Specifically, performing PCA on the collected data, we can identify the number of dominant synergies as a function of variation in the mapping."

We hope this provides more informed avenues for future empirical testing of our framework and the hypotheses we have put forth within.

Comments by Reviewer # 2

[R2: 1] "The work is defenitely of interest for the scientific community, the model proposed by the authors capable of continuously reproduce the behaviour of subjects while dealing with learning a task in hihgdimensional space is interesting."

We are grateful to the reviewer for their thoughtful feedback. We deeply appreciate the time and effort they put into reviewing our manuscript and offering suggestions for improvement. We have strived to address their concerns and comments as thoroughly as possible and hope that the manuscript is now clearer and more refined.

[R2: 2] "I like when at the end of the introduction the goal and the hypothesis of the work are presented. Please adjust the text accordingly. "

We thank the reviewer for this comment, and we understand the utility in clearly stating the goal of the study at the end of introduction. We have modified the 'Introduction' and included the following at the end to reflect the changes (page 2)-

"The goal of this work is to study human motor learning for high-dimensional novel learning task through a computational model that can explain various motor learning phenomena. Toward this end, we first develop an integrated dynamic model of human motor learning through the formation of internal representation, including models for perception, and forward and inverse learning. We use the motor synergies extracted from the human hand postures to create low-dimensional learning states, thus tackling the issue of increasing computational complexity with increasing DoFs of motor systems. We establish convergence properties of the proposed model and after fitting human participant data, we show that the proposed model can explain human motor learning and output performance behavior well. We then use the proposed model to systematically investigate the influence of model parameters on several motor learning trade-offs, including, speed-accuracy, exploration-exploitation, satisficing, and flexibility-performance. This analysis reveals how the motor system optimizes the use of synergies to control large degrees of freedom, how they manage various learning trade-offs, and how satisficing behavior is observed in a motor learning setting."

[R2: 3] "The section "moter learning experiment" should provide a little bit more of details so to allow the user to understand better the results. I know that the methods are at the very end of the manuscript but the reader needs help before the results. For example it is not clear if the task is a center-out task o a random walk. Also, how is the matrix C obtained? it is the same for all participants or each subject has a specific C? Only in the method I read about PCA for example."

We thank the reviewer for this comment, and we realize how the lack of sufficient experiment details can inhibit the understanding of the results.

For clarification, the task is neither a random walk nor a center out task. The sequence of targets was randomized in each session but it always consisted of 12 center out movements to each of the 3 outer

targets from the center target, and 24 movements to the center target (8 from each of the 3 outer targets). The matrix C is obtained through PCA on the data collected during the calibration phase and thus is different for each participant.

We have expanded upon the section 'Motor learning experiment' to include all the necessary and sufficient details about the experiment paradigm. Following are the changes we have made (page 3)-

"Participants first engage in a calibration phase where they move their hand fingers randomly while avoiding any extreme range of motion. The corresponding hand finger posture data is collected through the data glove, centered, and then PCA is performed to extract the principal components (PCs). The first two PCs are used as the rows to design the projection matrix C specific to each participant. The game bounds are also specialized to each participant to make sure that all the points in the game window are reachable (refer to Methods for more details). During the target capture gameplay, the participants need to move the cursor to the prescribed target point one after the other displayed as the centers of the squares on a 5×5 square grid, and a new target is prescribed only after the current target is captured. The participants trained on 4 target squares (3 outer targets and 1 center target) for 8 sessions, each comprising of 60 target capture movements. The sequence of targets prescribed in each session was randomized but it always consisted of 12 center out movements to each of the 3 outer targets from the center target, and 24 movements to the center target (8 from each of the 3 outer targets). Participants were further instructed to try and capture the prescribed targets within 2s of the movement onset from the previously captured target position, failing which the target square would highlight in red to indicate that they had exceeded the time. Although there was no maximum time limit to capture the target, a scoring system based on the movement time and accuracy was shown on top of the game window for motivation purposes (refer to [2] for more details)."

[R2: 4] "Can you please comment about the fact that the subject is controlling the velocity of the cursor and not the position? This is what I understand reading the section about the Model before the Results. But conversely when reading the methods I understand exactly the opposite. There the authors talk about hand postures. What is the subjects controlling? position or velocity? What are the authors modeling? Position or velocity? This part is very important to clarify, because depending on that I might have some additional questions."

We thank the reviewer for the comment, and we now address this.

We would first like to emphasize that the joint position to cursor position mapping, and the joint velocity to cursor velocity mapping are equivalent as long as the mean postures are calibrated to the center of the 5×5 grid. We write the BoMI mapping as finger joint velocities being mapped to the cursor velocities (through the mapping C). Integrating the cursor and joint velocities over small time periods, the velocity mapping can be equivalently written as increment in joint positions being mapped to the increment in cursor positions. Correspondingly, the participants are controlling the increments in the cursor positions through increments in finger joint angle values. Additionally, since the mapping is linear, and the mapping matrix is time-invariant, the increment in positions add up over long time periods to give the equivalence relation between the joint position to cursor position mapping, and the joint velocity to cursor velocity mapping.

We would also like to point out that this setup is different from the experimental paradigm undertaken in other studies, for instance [3], where the joint (IMU) angles are mapped to the end-effector velocities of a robot arm being controlled.

We have included this clarification and distinction in the footnotes of the revised manuscript as follows (page 3)-

"Since the mapping is linear and the mapping matrix C is time-invariant, the joint velocity to cursor velocity mapping, the and joint position to cursor position mapping are equivalent, as long as the initial

cursor position is the same. Also note that this setup is different from the experimental paradigm undertaken in other studies, for instance [3], where the joint (IMU) angles are mapped to the end-effector velocities of a robot arm being controlled."

[R2: 5] "line 74 R has dimension hxm...what number is h?"

h here is the number of synergies that are used to decompose the forward mapping matrix $C = W\Phi$. These synergies are collected as the rows of the matrix Φ, obtained from PCA of the calibration data. The following in section 'Model' contains the clarification (page 3) -

"We, therefore, decompose the mapping matrix $C = W\Phi$, where $\Phi \in \mathbb{R}^{h\times m}$ is a matrix of h basic synergies underlying coordinated human finger motions, and $W \in \mathbb{R}^{n\times h}$ represents the contributions (weights) of these synergies (see *Methods: Extracting motor synergies* for details on how the synergy matrix Φ is formed)."

[R2: 6] "line 105. Why 2 seconds? what is the maximum time available to the subject for reaching a target? please add this detail in the previous part where the task is presented."

The data used here is taken from the previous study [2], where the participants were instructed to try and capture the targets within 2 seconds of movement time (failing which the target would highlight in red to indicate that the participant had exceeded the time). Thus, consistent with the previous study we compute the reaching error at 2 seconds. Additionally, there was no maximum time limit for the participants to capture the targets.

We have included these details in the section 'Motor learning experiment' (page 3),

"Participants are further instructed to try and capture the prescribed targets within 2s of the movement onset from the previously captured target position, failing which the target square highlights in red to indicate that they have exceeded the time. Although there is no maximum time limit to capture the target, a scoring system based on the movement time and accuracy is shown on top of the game window for motivation purposes (refer to [2] for more details).",

and in 'Methods: Experiment procedure' (page 15) in the manuscript-

"Participants are additionally instructed to reach the center of the targets within 2s of the movement start, failing which the target block highlights in red and participants incur a time penalty on the score. This scoring system is shown at the top of the game window which does not serve any functional purpose other than motivating the participants."

We thank the reviewer for pointing out this omission, and we hope the manuscript is clearer now.

[R2: 7] "It would be nice to see FME curves for all 6 subjects."

We have included the `FME` curves for all 6 subjects in the revised manuscript in Fig 2 (page 6). We have also included them below for quick reference-

[R2: 8] "line 351-352. Can you please comment more on this hypothesis?"

We thank the reviewer for this comment, and we now provide some clarification.

The optimality parameter μ included in the cost function (Eq (7)) allows us to weigh the contribution of cost incurred in the task space versus cost incurred in joint velocities. Similarly, in the study by Danziger *et al.*[1], we can include μ in our HML model to capture trade-off between the task objective (capturing the target with the robot end effector), and minimizing the total change in the joint angles of the two-link arm during a target capture. The value of μ obtained from the data fitting procedure would then capture the effect observed in the study [1]. Specifically, a higher value of μ should imply higher optimality in terms of minimizing the total change in the two-link arm joint angles.

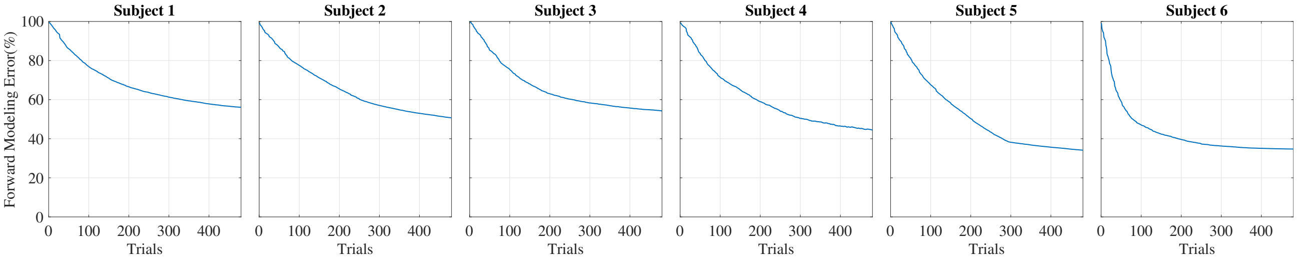

Figure 1: **Forward Model Error.** Evolution of forward model error (FME) for the fitted model as a function of trials for all 6 subjects.

We have made the following updates to the manuscript in section "Discussion: HML model and its parameters" to reflect this clarification (page 13)-

"Danziger *et al.* [1] concluded that, as opposed to participants moving the cursor on the screen along straight lines to achieve the task if the cursor is represented as the endpoint of a two-link arm, participants tend to minimize the distance in terms of joint angles of the two-link arm. The second objective that minimizes the total change in two-link arm joint angles during a target capture can be included in the cost function (7) to derive the inverse learning dynamics. The corresponding parameter value μ, that weighs the trade-off between the task objective (capturing the target with robot end-effector) and minimizing the change in two-link arm joint angles, would capture this effect observed in the study. A higher value of the optimality parameter would thus correspond to the participants trying to minimize the total joint angle changes during a target capture more strongly. However, further investigation is warranted to support this hypothesis, which is beyond the scope of the current work."

[R2: 9] "In the abstract "Conventional approaches to enhancing..." I believe this should be "to enhance..." lines 23 and 29, 169 the word "Ref" should not be present, the number between brackets is enough. Fig 2 caption. I believe the authors used wrong panel labels. From what I see in the Fig. data from human experiment are (b) and (d). Also in the caption the acronym "FME" used in the y label of panel (e) is missing."

We thank the reviewer for pointing out the errors and points for improvement. We have incorporated the suggestions in our revised manuscript.

[R2: 10] "Fig 6. What is the unit of measure of ρ?"

ρ values are relative to the size of the unit cell of the 5×5 game grid. For example, $\rho = 0.3$ means the radius of the target is 30% of the side of the unit cell. We thank the reviewer for pointing this out and we have included the following clarification in the Fig. 7 caption (page 10)-

"Values of ρ are relative to the size of the unit cell of the game grid."

References

[1] Zachary Danziger and Ferdinando A Mussa-Ivaldi. The influence of visual motion on motor learning. *Journal of Neuroscience*, 32(29):9859–9869, 2012.

[2] Rajiv Ranganathan, Adenike Adewuyi, and Ferdinando A Mussa-Ivaldi. Learning to be lazy: Exploiting redundancy in a novel task to minimize movement-related effort. *Journal of Neuroscience*, 33(7):2754–2760, 2013.

[3] Rajiv Ranganathan, Mei-Hua Lee, Malavika R. Padmanabhan, Sanders Aspelund, Florian A. Kagerer, and Ranjan Mukherjee. Age-dependent differences in learning to control a robot arm using a body-machine interface. *Scientific Reports*, 9(1):1960, 2019.

[4] Marco Santello, Martha Flanders, and John F Soechting. Postural hand synergies for tool use. *Journal of Neuroscience*, 18(23):10105–10115, 1998.
